# Insights into vitamin A in bladder cancer, lack of attention to gut microbiota?

**DOI:** 10.3389/fimmu.2023.1252616

**Published:** 2023-08-29

**Authors:** Peiyue Luo, Liying Zheng, Junrong Zou, Tao Chen, Jun Zou, Wei Li, Qi Chen, Biao Qian

**Affiliations:** ^1^ The First Clinical College, Gannan Medical University, Ganzhou, Jiangxi, China; ^2^ Department of Urology, The First Affiliated Hospital of Gannan Medical University, Ganzhou, Jiangxi, China; ^3^ Key Laboratory of Urology and Andrology of Ganzhou, Ganzhou, Jiangxi, China; ^4^ Department of Graduate, The First Affiliated Hospital of Gannan Medical University, Ganzhou, Jiangxi, China

**Keywords:** vitamin A, retinoic acid, gut microbiota, lipopolysaccharides, bladder cancer

## Abstract

Vitamin A has long been associated with bladder cancer, and many exogenous vitamin A supplements, vitamin A derivatives, and synthetic drugs have been investigated over the years. However, the effectiveness of these strategies in clinical practice has not met expectations, and they have not been widely adopted. Recent medical research on intestinal flora has revealed that bladder cancer patients exhibit reduced serum vitamin A levels and an imbalance of gut microbiota. In light of the close relationship between gut microbiota and vitamin A, one can speculate that a complex regulatory mechanism exists between the two in the development and occurrence of bladder cancer. As such, further exploration of their interaction in bladder cancer may help guide the use of vitamin A for preventive purposes. During the course of this review, attention is paid to the influence of intestinal microbiota on the vitamin A metabolism and the RA signaling pathway, as well as the mutual promotion relationships between them in the prevention of bladder cancer, In addition, it emphasizes the importance of intestinal microbiota for bladder cancer prevention and treatment.

## 1 Introduction

There has been an increase in the incidence of bladder cancer in recent years, particularly among women. It affects the urinary system and can be fatal. In 2020, it was reported to account for 573,278 new cases and 212,536 deaths worldwide, ranking ninth and 13th in terms of incidence and mortality among malignant tumors, respectively (1). Bladder cancer includes a variety of pathological types, including urothelial carcinoma, squamous cell carcinoma and adenocarcinoma, among which urothelial carcinoma is the most common pathological type (2). In reality, approximately 75% of patients with bladder cancer are afflicted with non-muscle invasive bladder cancers (NMIBC), which can have varying levels of risk for recurrence and progression. Generally, the 5-year survival rate for NMIBC exceeds 90%, contributing to the high incidence and low mortality rates of bladder cancer. Nonetheless, most NMIBC patients require long-term surveillance and preventive interventions, such as cystoscopy, which significantly impact their quality of life and impose a financial burden (3, 4). Therefore, chemoprophylaxis and other strategies to reduce postoperative bladder cancer recurrence have been widely employed in clinical practice, with retinoic acid (RA) being the most commonly used chemoprophylaxis drug. RA possesses remarkable anti-tumor properties. As early as 1990, it was discovered that RA could arrest hematopoietic cell cycle and induce cell differentiation into hematopoietic terminal cells, leading to its application in treating acute promyelocytic leukemia (5). Other cancer types, such as thyroid and prostate cancer, have also been shown to respond to RA’s anti-tumor effects, including inhibition of cell proliferation and induction of cell differentiation (6, 7). Vitamin A has been shown to prevent and treat bladder cancer in numerous studies conducted over the past 50 years. A review of these studies on vitamin A and bladder cancer is presented in **Table 1**. Researchers have confirmed that patients with bladder cancer have lower levels of serum vitamin A than healthy people, as shown in a substantial body of research. In addition, low vitamin A levels are increasingly regarded as risk factors for bladder cancer. Several *in vivo* experiments on vitamin A are summarized in **Table 2**. Almost all studies have shown positive results, mainly manifested as vitamin A can inhibit apoptosis, reduce tumor size, and inhibit the progression of bladder cancer. However, although *in vivo* experiments showed consistent promising results, the results of several clinical trials of vitamin A supplementation were not as expected (19–21). As a result, enhancing the efficiency of vitamin A in bladder cancer prevention and treatment would be an important research endeavor.

**Table 1:** Clinical trials investigation on vitamin A and bladder cancer.

Country	Study period	Age(years)	Case/subjects	Pathology	Main Findings	Reference
Sweden	1985 to 1987	40 to 74	418/929	urothelial carcinoma	Vitamin A supplement plays a certain preventive effect on urothelial carcinoma.	(8)
Eypt	1957 to 1965	Not mentioned	70/144	Not mentioned	Vitamin A levels were significantly lower in bladder cancer patients with squamous cell carcinoma than in normal individuals	(9)
Japan	1990 to 2007	>40	42/1666	urothelial carcinoma	High serum carotene levels reduce the risk of bladder cancer	(10)
USA	1957 to 65	40 to 89	-/8606	Not mentioned	Dietary vitamin A is associated with a reduced risk of squamous epithelial carcinoma	(11)
Turkey	Not mentioned	40 to 79	23/91	urothelial carcinoma	Compared to the control group, patients had significantly lower serum vitamin A levels	(12)
USA	2001 to 2004	30 to 79	1418/2589	urothelial carcinoma	Elevated plasma carotene levels significantly reduce the risk of bladder cancer	(13)
USA	1993 to 2007	45 to 75	581/185885	Not mentioned	Women with high vitamin A and carotene intake have a lower risk of bladder cancer.	(14)
USA	1971 to 1995	52 to 71	111/222	urothelial carcinoma	No significant correlation between serum carotene levels and bladder cancer risk after adjustment with smoking.	(15)
Japan	1971 to 1975	Not mentioned	27/6800	Not mentioned	Serum vitamin A levels were not associated with bladder cancer risk	(16)
Belgium	1999 to 2004	Not mentioned	178/540	urothelial carcinoma	Retinol intake was not significantly associated with bladder cancer.	(17)
Netherlands	1981 to 1989	55 to 69	569/3692	Not mentioned	There was no association between bladder cancer and dietary or supplemental intake of vitamin A and most carotenoids	(18)
USA	1981 to 1989	Not mentioned	335/11580	Not mentioned	Supplementing with β-carotene and vitamin A did not reduce bladder cancer risk significantly	(19)
USA	1980 to 2000	30 to 55	237/88796	Not mentioned	Vitamin A and carotene intake were not associated with bladder cancer risk.	(20)
USA	2000 to 2007	50 to 76	330/77050	urothelial carcinoma	Supplementation of carotene and retinol cannot effectively prevent the occurrence of urothelial carcinoma.	(21)

**Table 2:** *In vivo* studies investigating the effects of vitamin A in animal models of bladder cancer.

*In Vivo* Model–Carcinogen	Species	Outcome	Reference
MNNG	Rat	Bladder cancer incidence induced by MNNG was higher in rats with low vitamin A diet	(22)
FANFT	Rat	Vitamin A deficiency accelerated the carcinogenic efficiency of FANFT, but high vitamin A did not significantly inhibit FANFT-induced bladder cancer	(23)
BBN	Rat	Hyperretinemia inhibited the incidence of BBN-induced transitional cell carcinoma and neoplasms of the bladder	(24)
BBN	Rat	Vitamin A diet could reduce the progression of early bladder cancer by reducing BBN-induced urothelial atypia.	(25)
BBN	Rat	Vitamin A supplementation reduce the incidence of tumor and tumor size.	(26)
BBN	Mouse	Vitamin A treatment reduce the urothelial atypia and apoptosis in early bladder cancer.	(25)

MNNG, N-methyl-N’-nitro-N-nitrosoguanidine; FANFT, N-[4-(5-nitro-2-furyl)-2-thiazolyl] formanmide; BBN, N-butyl-N-(4-hydroxybutyl) nitrosamine.

Intestinal microbiomes are collections of microorganisms found in the gastrointestinal tract. Advancements in technologies such as 16S rRNA sequencing have revealed the significant role of gut microbiota in non-infectious diseases, particularly in tumor diseases. It is becoming increasingly clear that gut microbiota influences immunity and inflammation in intricate ways, implying its complex involvement in tumor occurrence and development (27). In addition, studies have reported enhanced anti-cancer effects associated with gut microbiota (28). Consequently, there is increasing attention on the role of gut flora in cancer. In a case-control study, bladder cancer patients’ gut microbiota was compared to that of healthy individuals. According to the findings, patients with bladder cancer showed a significant reduction in the abundance of specific bacteria in their gut. Additionally, this work used real-time qPCR to analyze the differences among 12 major Firmicutes, Bacteroidetes, Actinobacteria, and Proteobacteria bacteria. The findings demonstrated that the numbers of domain Bacteria, Clostridium cluster XI and Prevotella in patients were significantly lower than those in healthy group (29). Some studies (30–32) have achieved positive results through the application of intestinal probiotics such as Bifidobacterium pseudolongum, Lactobacillus johnsonii and Lactobacillus rhamnosus preparation in the prevention and treatment of bladder cancer. We have observed that the abundance of some of these probiotics such as Lactobacillus in the intestine is closely related to RA, either promoting or inhibiting (33, 34). So, the relationship between RA, gut flora, and bladder cancer is very subtle, and it is therefore necessary to further explore the interaction between these three factors in order to gain a deeper understanding.

## 2 Vitamin A metabolism and its role in bladder cancer

### 2.1 Absorption, transport and metabolism of vitamin A

**Figure 1:**
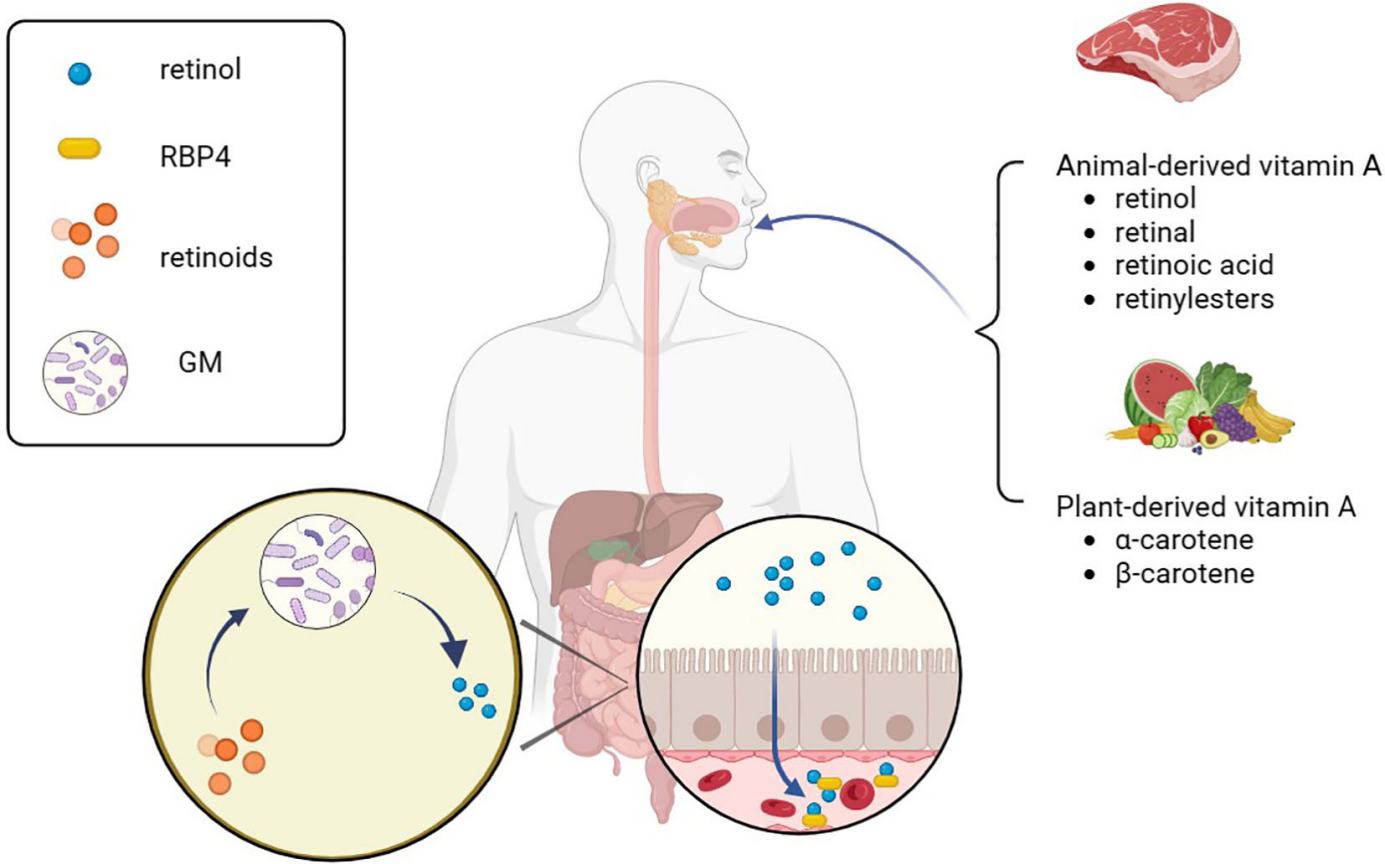
Vitamin A absorption. Various animal-derived vitamin A is first converted into retinol in the gastrointestinal tract, then absorbed into the blood through the intestine, combined with RBP4 for transport in the blood, and finally transported to the liver for storage. A part of phytogenic vitamin A can be converted into retinol by β-carotene oxygenase and absorbed, and the other part can be integrated into chylomicron with retinol and enter the blood circulation through lymphatic reflux. RBP4, retinol-binding protein 4; GM, gut microbiota.

The human body lacks the ability to directly synthesize RA, so we primarily obtain it from our diet. It is possible to absorb preformed vitamin A directly from animal foods, such as liver or fish, in the form of retinol, retinal, RA, and retinyl esters, which can be directly absorbed into the bloodstream by the gut and stored in the liver (35). However, for our bodies, food sources rich in β-carotene are the primary source of vitamin A. In the gastrointestinal tract, β-carotene is broken down and released, subsequently converted into retinal and retinol with the help of β-carotene oxygenase (BCO) (36). It then binds to retinol-binding protein 4(RBP4) and is transported through the bloodstream to the liver for further metabolism. In addition, retinoids and β-carotenes can be directly absorbed from food, packaged as chylomicrons, and enter the bloodstream through the lymphatic system (37) (**Figure 1**).

Target cells take up retinol that binds RBP4 from the blood through retinoic acid 6 (STRA6). Alternatively, retinol and β-carotenes from chylomicrons are taken up by target cells through lipoprotein-specific receptors (38). After entering the target cells, β-carotenes are converted to retinol by BCO. However, retinol has poor water solubility, so to enhance its transportation within cells, it binds with cellular retinol-binding proteins (CRABPs), which helps it exert its metabolic activity more effectively (39). Then, retinol is converted into RA through the action of retinol dehydrogenases (RDHs) and aldehyde dehydrogenases (ALDHs) (37, 40). The enzyme lecithin retinol acyltransferase (LRAT) finally converts RA and retinol into esterified products (36). RA, being the most active molecule among retinoids and the primary component of metabolically active vitamin A, activates the RA signaling pathway, which controls cell proliferation, differentiation, and apoptosis (41). It can be oxidized into non-biologically active compounds by the enzyme cytochrome P450 (CYP26) or transported to the nucleus by binding with CRABP or fatty acid binding protein 5 (FABP5) to activate the RA signaling pathway and exert its biological activity (42, 43).

**Figure 2:**
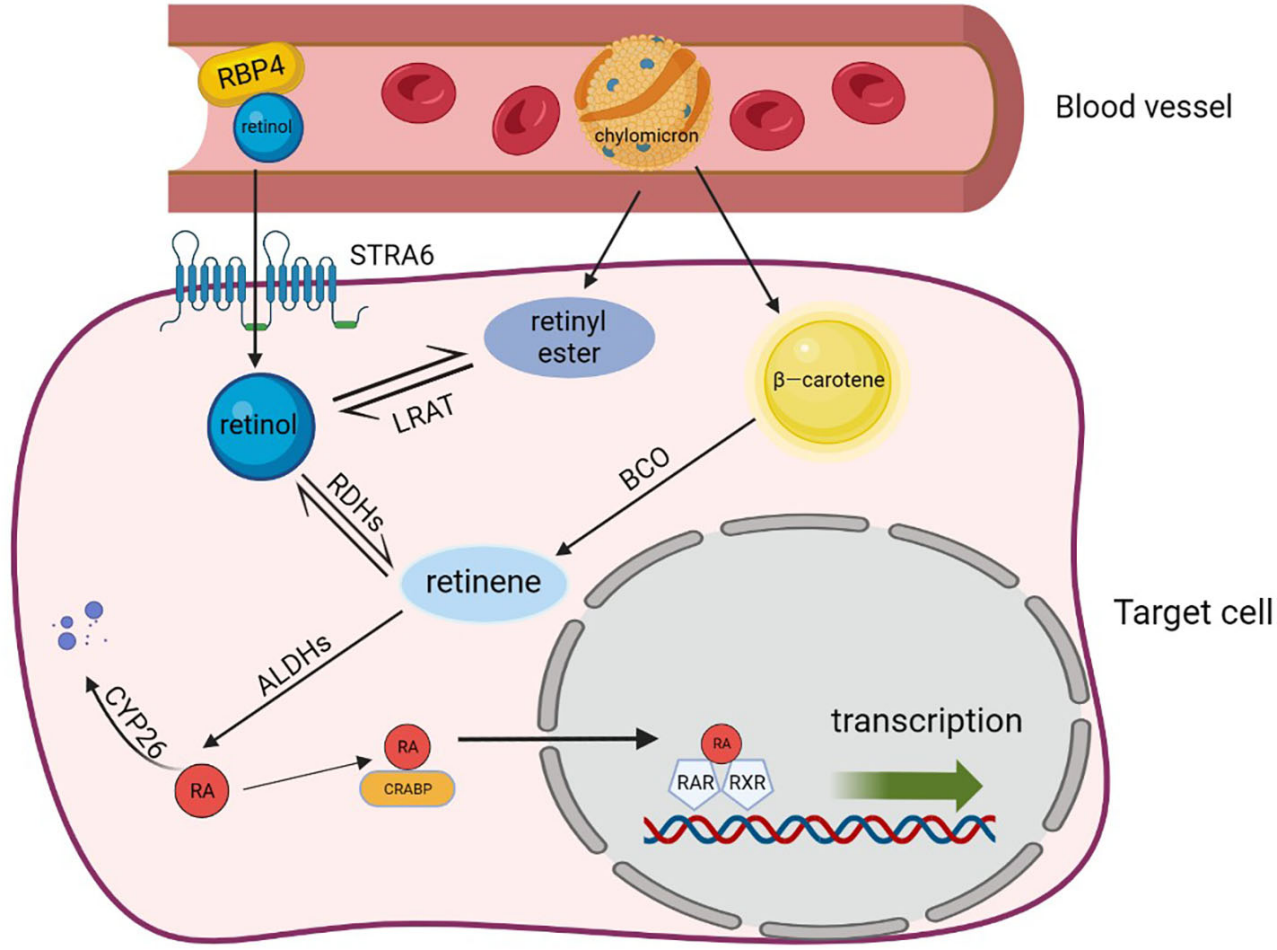
Retinoic acid metabolism. Retinol, β-carotene and chylomicron enter target cells through specific receptors and are transformed into the most active RA after a multi-step enzymatic reaction. RA binds different protein transporters and then interacts with different receptors. It also binds to CRABP and is transported to the RAR/RXR dimer to activate transcription, which can regulate the expression of genes such as cell proliferation and metabolism. In addition, RAs that are not transported to the nucleus are eventually degraded by CYP26 and lose functional activity. RBP4, retinol-binding protein 4; STRA6, stimulated by retinoic acid gene 6 protein; LRAT, lecithin retinol acyltransferase; RDHs, retinol dehydrogenases; ALDHs, aldehyde dehydrogenases; RA, retinoic acid; FAB5, fatty acid binding protein; CRABP, cellular retinol-binding proteins; PPAR, peroxisome proliferator activating receptor; RAR, retinoic acid receptor; CYP26, cytochrome P450 family 26.

RARs are classified as members of the steroid/thyroid hormone nuclear receptor superfamily, and RXRs are their indispensable eheterodimerization partners, all of which exist in the form of three para-homologs (RARα, RARβ, and RARγ as well as RXRα, RXRβ, and RXRγ) (44). There are more than 500 genes currently dependent on RA signaling, and activation of different isomers can lead to different biological effects (45–47). After entering the nucleus through CRABP, RA binds to RAR-RXR heterodimers and affects gene expression, which can be described as a molecular switch (48). RARs attach to the co-repressors NcoR1 and NcoR2 when RA is absent, and the co-repressors serve as bridges to connect a polymer complex with histone deacetylase activity (49). The complex has the ability to remove the acetyl group from the end of the histone to retain the chromatin’s condensation state and prevent the target gene from being transcribed. Conversely, co-repressors are dissociated from the RAR-RXR heterodimers and replaced by the co-activators such as nuclear receptor co-activator (NcoA1, NcoA2, and NcoA3) when RA binds to RAR. These co-activators may acetylate lysine residues in histone H3 and H4 or act as a platform to let other proteins or complexes on DNA change dynamically and rebuild nucleosomes (50). Finally, RA triggered modification of the chromatin, activation of the transcription machinery, and transcription of the target gene [**Figure 2**, adapted from Tratnjek et al. (51)]. Furthermore, it has been reported that after being transported to the nucleus through FABP5, RA can also bind to the peroxisome proliferator activating receptor (PPARβ/δ) to regulates the expression of genes that control cell proliferation, metabolism, and other vital functions (52, 53). But this conclusion is still controversial.

### 2.2 The effect of RA signaling pathway in bladder cancer

It has been demonstrated that RA induces the differentiation of mouse embryonic stem cells (ESC) into urinary tract epithelial cells in a vitro environment (53). Gandhi et al. (54) emphasized the crucial role of RA in maintaining adult urothelial homeostasis and confirmed the involvement of the RA signaling pathway in urothelial specification, homeostasis, and regeneration. Their study suggested that RA synthesized in stromal compartments acts as a critical regulator of urothelial maintenance through Wnt, Bmp, and Shh signaling. Moreover, RA also plays a significant role in tumor invasion and migration. A study (55) revealed that RA effectively inhibits the expression of matrix metalloproteinase-13 (MMP-13) mRNA, which is known to promote tumor invasion and metastasis by enhancing extracellular matrix degradation during tumor growth. Based on Wang et al. (56) experiments, he demonstrated that synthetic RA 4-HPR increases E-cadherin expression, as well as increased cell adhesion, promoting its translocation to the nucleus and inducing epithelioid cell transformation while reducing cell invasion capability. Additionally, RA can reverse epithelial-mesenchymal transition (EMT) and inhibit the invasion and migration of bladder cancer cells.

**Table 3** [adapted from Tratnjek et al. (51)] summarizes the *in vitro* study results of retinoids in bladder cancer cell lines. The RA plays a significant role in the proliferation, differentiation, migration, and invasion of bladder cancer cells, which makes it a key player in the disease’s development and progression. As a result, low serum levels of carotene and retinol are often seen among bladder cancer patients (9, 10, 12). In light of the important role of the retinoid signaling pathway in bladder cancer, restoring retinoid function could be a potential therapeutic option to prevent and treat bladder cancer. The different signal transduction pathways in retinoids have been shown to interfere with cell cycle progression in a variety of human cancer cells, particularly by regulating cyclins, CDKs, and cell cycle inhibitors (64, 65). Wang et al. (66) co-cultured RA with bladder cancer cells EJ and found that RA could significantly inhibit the growth of bladder cancer cells and reduce the expression of mutant P53 in cells. Zou et al. (63) evaluated the effects of three types of retinoids, namely all-trans-retinoic acid (ATRA), N-4-hydroxyphenyl-retinamide (4-HPR), and 6-[3-(1-adamantyl)-4 hydroxyphenyl]-2-naphthalene carboxylic acid (CD437), on the growth, apoptosis, cell cycle, and receptor expression of bladder cancer cells. They found that cells exposed to all three retinoids exhibited varying levels of apoptosis, G1 cell cycle arrest, and growth inhibition. Numerous animal experiments have also evaluated the chemoprophylaxis and treatment effects of RA on animal models of bladder cancer. Among the carcinogens, N-butyl-N-(4-hydroxybutyl)-nitrosamine (BBN) is commonly used, as it is closely related to certain carcinogens found in tobacco smoke and exhibits remarkable bladder specificity (67, 68). Several studies (26, 69) have demonstrated that RA can significantly reduce urothelial atypia and apoptosis, decrease the incidence of urothelial carcinoma, and effectively inhibit BBN-induced urothelial carcinoma.

**Table 3:** The effects of retinoids in bladder cancer cell lines.

Retinoids	Application	Molecular and phenotypic effects	Reference
ATRA	RT112	Inhibition of epidermal growth factor-induced cell proliferation.	(57)
	HT-1376	Inhibits cell proliferation by inhibiting the activity of related transcription factors	(58)
	T24	Inhibition of cellular retinol-binding protein-II expression Direct inhibition peroxisome proliferator-activated receptor PPARβ/δ	(59)
4-HPR	T24	Promote the expression of E-cadherin and promote the transfer of β-catenin from the nucleus to the cytoplasm	(56)
	RT4 UM-UC-9/10/14	Inhibition of cell growth and the induction of apoptosis	(60)
13-cis-RA	NHU	inhibition of squamous metaplasia and reverting to basal phenotype	(61)
ATRA CD437 4-HPR	RT4 T24 UM-UC-2/3/6/10/13/14	Induction of apoptosis and G1 cell cycle arrest, and the inhibition of cell growth	(62)
ATRA 9-cis-RA 13-cis-RA	RT4 T24	Inhibit the expression of matrix metalloproteinases	(55)

ATRA, all-trans retinoic acid; 4-HPR, N-(4-Hydroxyphenyl)-retinamide or fenretinide; 9-cis-RA, 9-cis-retinoic acid; 13-cis-RA, 13-cis-retinoic acid.

Taken together, the RA signaling pathway is implicated in developing and progressing bladder cancer, and the use of exogenous RA supplementation may prove to be an effective method of preventing bladder cancer occurrence as well as postoperative recurrence. However, the current clinical use of RA supplementation is limited due to variations in the expression and distribution of PPAR and RXR subtypes in the human urothelium and the potential toxic effects of vitamin A (69, 70). In addition, pharmacological applications of RA also have limitations, such as short half-life, poor water solubility, sensitivity to light, heat and oxidants, and rapid degradation during digestion, leading to low bioavailability and bioaccessibility (71, 72). Therefore, addressing these challenges is crucial for improving the effectiveness of RA supplementation.

## 3 Promoting effect of gut microbiota on RA pathway

Therefore, it is evident that vitamin A undergoes a complex series of pathways encompassing absorption, metabolism, RA production, and subsequent activation of the RA signaling pathway. Any disruption in these processes can potentially impact the efficacy of vitamin A. Consequently, this review provides a comprehensive overview of the influence exerted by intestinal flora on these intricate pathways.

### 3.1 Promotes RA absorption through bile acids

As mentioned above, various forms of vitamin A precursors are present in different foods, and in the small intestine, they are absorbed mostly at the proximal part. β-carotene from plant-based foods is absorbed by small intestinal epithelial cells through passive diffusion after forming micelles with bile acids and dietary fats (73). However, animal-derived retinyl esters must be converted to retinol by retinyl ester hydrolases (REHs) before being absorbed by intestinal cells, and they are not directly absorbed by the intestine as retinol esters in the intestinal lumen (74). The primary enzymes involved in retinol ester hydrolysis in the intestinal lumen include pancreatic triglyceride lipase (PTL), carboxyl ester lipase (CEL) and the intestinal brush border membrane enzyme phospholipase B (PLB) (75), among which PTL is the most important REH in the intestinal cavity (76). It has been shown that bile acid sequestrants can lower serum levels of total carotenoids in humans, and bile acids can enhance the activities of PTL and CEL enzymes, promoting the absorption of retinol and its derivatives from animal-derived foods. In addition to absorption by intestinal epithelial cells, retinoids and β-carotenes can also be incorporated into chylomicrons along with triglycerides, cholesterol esters, phospholipids, cholesterol, and proteins in the Golgi apparatus of intestinal epithelial cells. These chylomicrons are then transported into the lymphatic circulation and subsequently re-enter the bloodstream through the lymph system. This process is also influenced by bile acids, as impaired chylomicron excretion has been observed in the absence of bile acids (59). In a mouse model of chylomicron retention disease, severely impaired fats and vitamins A and E absorption were observed, along with significantly reduced growth rates (77). Therefore, it is essential for the digestion, absorption, and dissolution of fat-soluble vitamin A from food that the concentration of bile acids in the small intestine is high.

Cholesterol is the raw material used to synthesize bile acids, which are a class of cholenoic acids. By passing through the tubule membrane of the gallbladder, they are synthesized in the liver and secreted into bile. The duodenum releases cholecystokinin after eating, stimulating the contraction of the gallbladder, which releases bile acids into the intestinal cavity for digestion (78). Approximately 95% of bile acids are then reabsorbed into the ileum and return to the liver through the portal vein, where they are once again secreted into bile (79). However, a portion of bile acids (approximately 200 to 800 mg per day in humans) escapes reabsorption in the gut and reaches the colon, where they are further metabolized by the gut flora, resulting in the production of secondary bile acids with increased hydrophilicity (80, 81).

Bile acids are metabolized by the gut microbiota, leading to secondary bile acids after three modifications are completed: uncoupling, 7α-dehydroxylation, and differential isomerism. An enzyme known as bile salt hydrolase (BSH) works on bile acids conjugated with glycine or taurine, which is required to perform 7α-dehydroxylation. Song et al. (82) investigated individuals from 11 groups across six continents and reported that the classification and identification of intestinal bacteria BSHs differed in taxonomy and abundance of BSHs in the human intestinal microbiome. The decoupled free bile acids were further transformed into secondary bile acids by 7α-dehydroxylation by microorganisms. Michael et al. (83) analyzed the signaling pathway of cholic acid dehydroxylation and found that certain strains, such as Bacteroides, Clostridium, Escherichia, Eubacterium and Lactobacillus, with the core bai gene cluster could induce dehydroxylation. In addition, the molecular modification of bile acids by intestinal bacteria also includes differential isomerization, which is the main process to enrich the diversity of intestinal bile acids.

It can thus be concluded that gut flora can influence bile acid pool size and bile acid composition in secondary forms, as confirmed in a study by Swann et al. (84), who observed that bile acid diversity was significantly reduced in sterile or antibiotic-treated rats while taurine-binding bile acid abundance was significantly increased. Further comprehensive studies (85) on gut microbiota, bile acid and vitamin A metabolism revealed that remodeling or alteration of gut microbiota resulted in lower bile acid levels, consequently reducing the absorption of vitamin A. Moreover, these studies support the hypothesis that the entire gut flora has a role in vitamin A metabolism.

### 3.2 Gut microbiota affects RA content by influencing the content of Ra-related enzymes

The content of RA is not only influenced by intestinal absorption but is also closely related to the levels and activities of RA synthesizing and degrading enzymes, such as ALDHs and CYP26. ALDHs are primarily found in the liver and intestines and comprise three main subtypes, namely ALDH1A1, ALDH1A2 and ALDH1A3, among which ALDH1A1 is the most abundant (37). The main function of ALDH1A1 is to participate in the second step of retinol oxidation, which oxidizes the retinol transported into cells into RA. Comparatively, CYP26 also comprises three subtypes, namely CYP26A1, CYP26B1 and CYP26C1, among which CYP26A1 has the strongest catalytic activity and can degrade RA into inactive hydroxylated and oxidized derivatives (86). An *in vivo* study (42) on trans-retinoic acid and colon cancer found decreased ALDH1A1 and ALDH1A3 protein expression, while ALDH1A2 protein expression remained unchanged in colon cancer progression with alterations in the gut microbiome. In addition, CYP26A1 colon transcription levels increase 3-8 times during the progression of the disease. In addition, the decrease of ALDH1A1 and increase of CYP26A1 were also corrected to a certain extent after the recovery of gut microbiota with antibiotics. Another study (87) found that feeding mice with Bifidobacterium infantis 35624 increased ALDH content in dendritic cells of the intestinal tract, resulting in a further rise in RA content.

The above studies found that gut microbiota was strongly correlated with retinoic acid (RA) metabolic enzymes. Further investigations have shown that this effect is mediated through lipopolysaccharides (LPS), a microbial product and Gram-negative bacteria’s outer membrane component (88). LPS interacts with toll-like receptor 4 (TLR4), its natural immune receptor, leading to the activation of signaling pathways. TLR4 signaling cascades activate the PI3K/Akt and NF-κB signaling pathways, resulting in subsequent biological effects (89). In an *in vivo* study, CYP26A1 and CYP26B1 mRNA expression was significantly suppressed in the liver of rats treated with LPS of P. aeruginosa in the presence of RA. Furthermore, Song et al. demonstrated the induction of dysbiosis in a mouse intestinal model through LPS injection, followed by subsequent administration of LPS into chicken embryos which resulted in an upregulation of retinal dehydrogenase 2 (RALDH2) mRNA expression (90). Additionally, quantitative PCR analysis revealed decreased expression levels of cytochrome P450 enzymes, namely Cyp26a1 and Cyp26c1, which are involved in RA metabolism. Furthermore, the study examined antioxidant enzymes and found that LPS treatment up-regulated mRNA expression of antioxidant enzymes such as glutathione peroxidase (GPX1), catalase (CAT) and NAD(P)H quinone dehydrogenase 1 (NQO1). Based on these, the researchers proposed that LPS induces oxidative stress by activating TLR, thereby influencing the levels of RA metabolic enzymes. Another *in vitro* experiment (91) showed similar results and attributed the results to LPS activation of the NF-κB pathway.

In summary, the gut microbiota can increase RA content by promoting RA synthesis enzymes and inhibiting RA degrading enzymes. This action is likely the result of the interaction between bacterial LPS and TLR signaling. It is worth noting that LPS can be further converted into fat micelles in the gastrointestinal tract, promoting the absorption of β-carotene and retinol.

### 3.3 Gut microbiota promotes the conversion of β-carotene to retinol in the intestine

After consuming fruits or vegetables rich in β-carotene, the compounds undergo various physical and chemical metabolic processes in the digestive tract, such as chewing and fermentation. Some of them can be processed into chylomicrons, which enter the bloodstream and are eventually transported to target cells. Within the target cells, β-carotene is enzymatically broken down into retinol. Other β-carotenes must be converted into retinol within the intestine before being combined with RBP4 for absorption. In short, plant-derived β-carotene needs to be converted into retinol to exert its biological functions, and this conversion process is primarily mediated by BCO, which is a highly potent enzyme found in various tissues of mammals, including jejunal epithelial cells, intestinal mucosa, liver, kidney, lung and brain. There are three paralogs of BCO: 15,15’-β-carotene oxygenase (BCO1), 9’,10’-β-carotene oxygenase (BCO2), and RPE65 (92). In the context of liver and intestinal tissues, BCO catalyzes the cleavage of β-carotene, splitting it in the middle to produce two retinal molecules. These retinal molecules are further oxidized to form RA (93).

As early as 1998, Grolier et al. (94) studied the biotransformation of carotenoids into retinoids in rat intestines and investigated the relationship between their bioavailability and the abundance of intestinal flora. Their results suggested that gut microbiota might influence absorption of carotenoids and retinoids, as well as their bioactivities. However, there have been no studies confirming the direct regulation of BCO by the gut microbiome. So far, only a metagenomics study (95) identified a gene in the human gut genomic library that shares homology with BCO. In a subsequent metagenomic study (96) of the human gut, certain Gram-positive and Gram-negative bacteria were found to possess brp/blh genes encoding bacteriorhodopsin-related protein-like homolog protein (Blh) and bacterioopsin-related protein (Brp), which is not homologous to BCO but has similar activity to BCO. Further experiments (97) were conducted to construct strains with brp/blh gene deletions, and the retinoid levels in the medium were measured. The results demonstrated that β-carotene levels were 3.8 times higher and retinol levels were 3.7 times lower than the wild type, thus validating the genomic prediction results. In recent years, brp/blh genes have been reported in proteobacteria, including Sphingopyxis alaskensis, Novosphingobium aromaticivorans and mycobacteria such as Mycobacterium tuberculosis (98). These genes encode enzymes that convert β-carotene into retinal, which could explain the role of the gut microbiota in facilitating vitamin A metabolism. Therefore, the gut microbiota may enhance the efficiency of vitamin A absorption from food by converting β-carotene to retinal in the gut or liver and encoding enzymes that exhibit similar effects to BCO, which may thus enhance the effect of RA downstream.

### 3.4 Regulating the expression and activity of RAR/RXR

Once transported into the nucleus by CRABP, RA exerts its signaling function by binding to various nuclear receptors and regulating downstream gene transcription. These nuclear receptors include RARs α, β, and γ, RXRs α, β and γ, and PPAR β/δ. In an investigational study (99), the mRNA expression of RAR was compared between normal and malignant bladder tissue specimens from human patients. The findings revealed a significant reduction in the level of RAR mRNA, particularly RARβ2 mRNA, among individuals with bladder cancer. RARs serve as substrates for various serine/threonine kinases, including PKA, PKC, and CDK7, which can phosphorylate them. The level of phosphorylation significantly impacts the activity of RARs. A study (100) revealed that Akt interacts with RARα and phosphorylates its DNA binding domain at Ser (90) residue, leading to a significant inhibition of its activity. Thus, the number and activity of RA receptors are also crucial for proper RA signaling.

Notably, the gut microbiota has been found to be closely associated with the content and activity of these nuclear receptors. Yuan et al. established an animal model of gut microbiota dysbiosis through administration of antibiotic mixtures to mice and observed a significant increase in serum IGF-1 levels due to dysbiosis-induced elevation of SCFAs. Consequently, this led to the activation of the IGF-1/Akt pathway and subsequent regulation of RAR phosphorylation. suggesting that SCFAs inhibit the RA response by enhancing RAR phosphorylation through the IGF-1/Akt pathway in cases of disrupted intestinal microecology (101). Moreover, additional studies (90, 91), by exposing chicken embryos to dysbacteriosis-derived LPS, revealed that intestinal microbiota can influence RA receptor activity through SCFAs and affect RA receptor expression through LPS induction. qPCR data showed that the mRNA levels of RAR (α, β, γ) and RXR (α, β, γ) in the cells were significantly changed after exposure to LPS. Another *in vitro* study (102) showed that the mRNA expressions of RARα and RARγ in hepatic stellate cells (HSCs) were significantly decreased following LPS treatment with an autophagy regulator. The expression of RARα and RARγ was restored after pretreatment with autophagy inhibitors, confirming that LPS may reduce RA receptor levels by activating autophagy.

## 4 Potential effect of gut microbiota on bladder cancer, also related to vitamin A

In summary, the intestinal microbiota plays a significant role in the absorption, synthesis, degradation, and regulation of retinoic acid (RA) and its receptors, highlighting the critical role for gut microbiota in RA signaling. However, the impact of the gut microbiota extends beyond RA metabolism. Emerging research has revealed that the gut microbiota can influence bladder cancer through various mechanisms. It is important to note that retinoic acid can also intervene in the anti-tumor effects mediated by the intestinal microbiota. This further reveals the close relationship among gut microbiota, retinoic acid, and bladder cancer, highlighting the critical role of the gut microbiota in this context.

### 4.1 Effects of gut microbiota on tumor

Intestinal microbiota refers to the trillions of microorganisms, including phages, viruses, bacteria, protists, worms and fungi, that colonize the intestinal tract. According to statistics, about 3.8 × 1013 bacteria colonize the intestinal tract, mainly comprising bacteroidetes and actinomyces (103, 104). Advances in 16SRNA gene sequencing and bioinformatics analysis have deepened our understanding of the intestinal microbiota, revealing its crucial role in human physiology and health (105). In addition to facilitating the digestive process and helping the body absorb nutrients from food (106), the gut microbiome also influences host metabolism (107), produces antibacterial substances that mediate the integrity of the intestinal barrier to protect the host from pathogens (108), and regulates host immunity to aid in the removal of harmful substances from the gut (109). In a number of studies (110, 111), it has been shown that gut microbiota diversity plays a critical role in human health. Under physiological or pathological conditions, an imbalance or disruption in the balance of the intestinal microbiome, known as gut microbiota disorder, can occur. Besides impairing the intestinal microbiome’s functions, this disorder may also contribute to cancer’s development and progression. For instance, an imbalance in the gut flora can cause inflammation that leads to colon cancer (112), and recent research on esophageal cancer similarly reported an imbalance in the flora (113).

**Figure 3:**
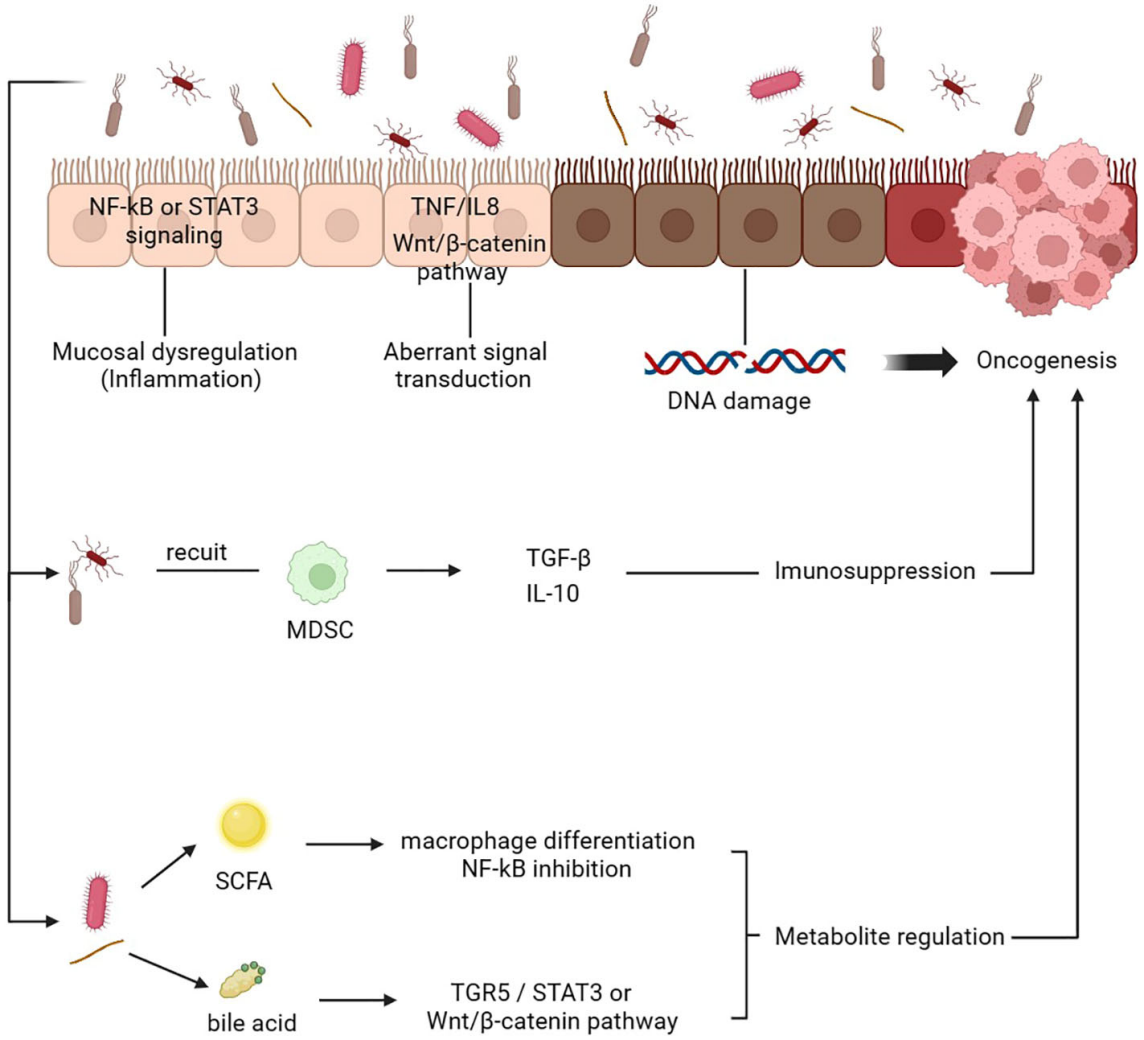
Mechanism of gut microbiota mediating tumor development. 1. The gut microbiota induces chronic inflammation by activating the NF-kB or STAT3 pathways and various tumorigenic-related pathways. 2. Abnormal signaling pathways TNF/IL-8 and Wnt/β-catenin promote the metastasis and invasion of tumor cells. 3. The gut microbiota induces DNA damage and cell proliferation. 4. Gut microbiota recruit MDSCs release active mediators, thus mediating immunosuppression and promoting tumorigenesis. 5. The gut microbiota can change the content of various metabolites. For example, SCFA can induce the differentiation of macrophages and inhibit the NF-kB pathway, while bile acids can activate multiple pathways to affect the tumor microenvironment and the occurrence and development of tumors. DC, dendritic cell; SCFA, short-chain fatty acid; MDSC, myeloid-derived suppressor cells.

It has been shown that a dysbiotic intestinal microbiota contributes to the onset and progression of cancer in several studies. The impact of intestinal microbiota on tumors can be categorized into several aspects. Firstly, it is thought that the gut microbiome contributes to tumor progression by inducing chronic inflammation and immunological responses. Through antigen presentation and activation of pattern recognition receptors, such as toll-like receptors, NOD-like receptors, and G-protein-coupled receptors, the intestinal microbiota can activate immunoinflammatory signaling pathways and influence inflammatory immune response (114). This imbalance of intestinal flora can regulate changes in inflammatory factors, thereby promoting tumor progression. For instance, certain intestinal bacteria may activate the NF-κB or STAT3 pathway to induce the production of cytokines such as IL-10 and IL-17, which are believed to promote tumor cell proliferation and metastasis (115). Secondly, gut microbiota produces specific metabolites, including short-chain fatty acids, tryptophan metabolites and secondary bile acids, which can either promote or inhibit tumor occurrence and development (116, 117). In colorectal cancer, intestinal secondary bile acids have been found to activate carcinogenic pathways such as TGR5/STAT3, WNT/beta-catenin, and NF-kB signaling, thus promoting tumorigenesis (116, 118, 119). Conversely, some short-chain fatty acids, particularly acetate, propionate, and butyrate, have been shown to inhibit the development of colorectal cancer. A recent meta-genomic and metabonomic analysis revealed decreased levels of butyrate-producing bacteria in colorectal cancer patients, suggesting the potential role of butyrate levels in colorectal cancer development (120). Furthermore, the gut microbiome can promote tumor development by causing DNA damage, promoting cell growth and apoptosis, and modulating the immune response. Notably, E. coli is a prominent example, as it can directly cause genomic instability and DNA damage (121) (**Figure 3**).

Furthermore, the impact of gut microbiota on tumors is also manifested in its influence on the tumor immune microenvironment. The tumor microenvironment (TME) serves as an internal milieu for the survival and proliferation of cancer cells, comprising various immune cells such as T lymphocytes, B lymphocytes, natural killer cells, and tumor-associated macrophages. The human immune system functions to conduct immune surveillance by identifying and eliminating abnormal cells; henceforth, tumors must evade or suppress this immunosurveillance to sustain their progression. TME is highly conducive to microbial invasion, colonization, and proliferation. A study (122) has demonstrated that intestinal microorganisms can migrate to the TME and induce immunosuppression. Similarly, Zhang et al. have confirmed that gut flora can prompt hepatocytes to recruit myeloid-derived suppressor cells (MDSCs) and produce tumor-promoting and anti-inflammatory chemicals such as TGF-β and IL-10, thereby establishing an immunosuppressive microenvironment that ultimately contributes to the development of cholangiocarcinoma (123). Furthermore, metabolites derived from gut microbiota also impede anti-cancer immunity. For instance, tryptophan metabolite produced by Lactobacillus, can activate aromatic hydrocarbon receptors in tumor-associated macrophages, thus inhibiting the infiltration of cytokines and immune cells in pancreatic cancer (124).

### 4.2 Effects of gut microbiota on bladder cancer, needs further exploration

It is worth noting that one study (125) found that immune cells, intestinal microbiota, metabolites, and cytokines can leave the intestinal tract through the blood circulation and induce corresponding pathological changes, indicating that the role of intestinal microbiota in promoting tumors may extend beyond the gastrointestinal tract to other areas, including bladder cancer. However, current studies have mainly focused on the relationship between bladder cancer and the urinary microbiome, leading to a relatively limited number of studies on the intestinal microbiome. Although it has been proposed that there might be a correlation between the intestinal microbiome and urinary microbiome, no direct comparisons have been made between the changes in the urinary and intestinal microbiomes in the same patient to confirm this conjecture (126). The most direct evidence linking the gut microbiota and bladder cancer comes from a study comparing the gut microbiota composition of bladder cancer patients with that of a normal population, which revealed alterations in the gut microbiota composition and significant differences in metabolite concentrations, such as butyric acid (29). In addition, the influence of dietary intervention on the intestinal microbial composition of mice on bladder cancer has been assessed in several *in vivo* experiments (28, 127, 128). The results demonstrate that normalizing the intestinal microbial composition through dietary intervention repairs the intestinal physiological barrier, reduces inflammation and immune response, inhibits bladder cancer progression, and enhances sensitivity to radiotherapy and chemotherapy. Lactic acid bacteria have been shown to be beneficial in avoiding the return of superficial bladder cancer in two clinical trials that compared their effectiveness to that of other biologics in preventing tumor recurrence following transurethral excision of bladder tumors (30, 129). These studies have offered preliminary evidence of the tight association between bladder cancer and gut microbiota, but further study is required to clarify the precise processes behind this association.

### 4.3 Auxiliary effects of vitamin A on tumor toxicity of gut microbiota

Interestingly, it has been observed that not only does the stable intestinal microbiota have a positive effect on the RA signaling pathway, but the level of vitamin A also has an important impact on the homeostasis of the intestinal microbiota, suggesting a mutually reinforcing positive feedback relationship. Micronutrient food sources, such as vitamins A, appear unlikely to have a significant impact on the gut microbiome, but studies have suggested that certain micronutrient signals, such as vitamin A, may first be amplified by inducing secretory mediators in intestinal epithelial cells and other stromal cells, leading to a stronger signal and influence on luminal microbes (130). This notion is supported by studies showing that vitamin A-deficient mice have impaired intestinal structural integrity and reduced Paneth cell numbers but increased secretion of goblet cells and mucins and that these secreted mucins, antimicrobial peptides and proteins have specific effects on the microbiome of these animals (131). Since mucin formation by goblet cells and low levels of antimicrobial peptides are two examples of how vitamin A deficiency affects the phenotypic and function of intestinal epithelial cells, it follows that these changes can impact the number and makeup of symbiotic bacteria in the gut. Studies have shown that vitamin A alleviates inflammation, enhances intestinal epithelial barrier function, and influences gut bacterial diversity *in vivo* (132, 133). Vitamin A deficiency leads to a specific reduction in the gut microbiome, ecological imbalance, impaired immune system function, and increased susceptibility to gastrointestinal infections or injuries (134). Mice treated with vitamin A or RA have shown higher gut microbiota diversity and altered bacterial composition (33, 135). Similar results have also been reported in clinical studies, which revealed significant differences in the gut microbiota composition among vitamin A intake groups (136). A study (34) on the stage-dependent effect of all-trans retinoic acid on lupus found that after two weeks of all-trans retinoic acid treatment, the abundance of intestinal lactobacillus decreased while clostridium increased, indicating that treatment with all-trans retinoic acid significantly altered the abundance of bacteria in the gut.

## 5 Conclusion

There is clear evidence indicating that the gut microbiota can enhance the absorption of RA by facilitating the transformation of vitamin A and influencing bile acid metabolism. It can also modulate the levels of RA by affecting key enzymes involved in RA synthesis and degradation. In addition, as summarized in the third part of the article, studies have also highlighted the potential impact of intestinal flora on bladder cancer through the production of specific metabolites or modulation of urethral microbiota, and RA has demonstrated a certain efficacy in modulating tumor-associated gut microbiota. Overall, the intestinal microbiota can contribute to the anti-tumor effects of the RA signaling pathway at multiple levels. However, direct evidence linking intestinal microbiota to enhanced inhibitory effects of RA in bladder cancer is currently lacking. Furthermore, the toxic effects of intestinal microbiota on bladder cancer have been demonstrated, and RA has been shown to play a significant role in the anti-tumor effects of the gut microbiota. The interaction between RA and the gut microbiome enhances the anti-tumor effects of each other. Consequently, any alterations in retinoic acid or the gut microbiota can disrupt this positive cycle, leading to an adverse feedback loop. Therefore, solely supplementing exogenous vitamin A may not provide optimal preventive effects for bladder cancer patients, as their gut microbiome may undergo alterations during the development and progression of the disease. Currently, synthetic retinoic acid drugs are being utilized in clinical practice to overcome the limitations of short half-life and poor patient tolerance. Encouragingly, these drugs have exhibited satisfactory therapeutic effects while maintaining good patient tolerability (137, 138). Additionally, promising feedback has been obtained from studies investigating intestinal probiotics (30–32). Although no reports exist regarding their combined application for bladder cancer treatment, it is reasonable to anticipate that further elucidation of the interplay between these three factors will pave the way for novel strategies in bladder cancer prevention and treatment.

## Author contributions

BQ conceived the manuscript, and PL performed the literature search and drafted the manuscript. JZ and TC edited tables and figures. WL and QC collected the data. JRZ and LZ reviewed and polished the manuscript. All authors have approved the final version submitted and agree to its submission to this journal.
