# Corrigendum: Insights into vitamin A in bladder cancer, lack of attention to gut microbiota?

**DOI:** 10.3389/fimmu.2025.1609154

**Published:** 2025-04-30

**Authors:** Peiyue Luo, Liying Zheng, Junrong Zou, Tao Chen, Jun Zou, Wei Li, Qi Chen, Biao Qian

**Affiliations:** ^1^ The First Clinical College, Gannan Medical University, Ganzhou, Jiangxi, China; ^2^ Department of Urology, The First Affiliated Hospital of Gannan Medical University, Ganzhou, Jiangxi, China; ^3^ Key Laboratory of Urology and Andrology of Ganzhou, Ganzhou, Jiangxi, China; ^4^ Department of Graduate, The First Affiliated Hospital of Gannan Medical University, Ganzhou, Jiangxi, China

**Keywords:** vitamin A, retinoic acid, gut microbiota, lipopolysaccharides, bladder cancer

In the published article “Tratnjek L, Jeruc J, Romih R, Zupančič D. Vitamin A and Retinoids in Bladder Cancer Chemoprevention and Treatment: A Narrative Review of Current Evidence, Challenges and Future Prospects. Int J Mol Sci. 2021 Mar 29;22(7):3510. doi: 10.3390/ijms22073510.” was not cited in the article. The citation has now been inserted in **Section 2. Vitamin A metabolism and its role in bladder cancer**, *Sub-section 2.1. Absorption, transport and metabolism of vitamin A*, Paragraph 3, “Finally, RA triggered modification of the chromatin, activation of the transcription machinery, and transcription of the target gene (Figure 2).” and should read:

“Finally, RA triggered modification of the chromatin, activation of the transcription machinery, and transcription of the target gene (Figure 2, adapted from Tratnjek et al.).”

And the citation has now been inserted in **Section 2. Vitamin A metabolism and its role in bladder cancer**, *Sub-section 2.2. The effect of RA signaling pathway in bladder cancer*, Paragraph 2, “Table 3 summarizes the *in vitro* study results of retinoids in bladder cancer cell lines. The RA plays a significant role in the proliferation, differentiation, migration, and invasion of bladder cancer cells, which makes it a key player in the disease’s development and progression.” and should read: “Table 3 (adapted from Tratnjek et al.) summarizes the *in vitro* study results of retinoids in bladder cancer cell lines. The RA plays a significant role in the proliferation, differentiation, migration, and invasion of bladder cancer cells, which makes it a key player in the disease’s development and progression.”

The authors apologize for this error and state that this does not change the scientific conclusions of the article in any way. The original article has been updated.

